# HDL Function Versus Small Dense LDL: Cardiovascular Benefits and Implications

**DOI:** 10.3390/jcm14144945

**Published:** 2025-07-12

**Authors:** Claudiu Stoicescu, Cristina Vacarescu, Dragos Cozma

**Affiliations:** 1 Cardiology and Cardiovascular Surgery Department, University of Medicine and Pharmacy Carol Davila, 37 Dionisie Lupu, 030167 Bucharest, Romania; claudiu.stoicescu@umfcd.ro; 2 Cardiology and Cardiovascular Surgery Department, University Emergency Hospital, 169 Splaiul Independenței, 050098 Bucharest, Romania; 3 Institute of Cardiovascular Diseases Timisoara, 300310 Timisoara, Romania; dragos.cozma@umft.ro; 4 Cardiology Department, Victor Babeș” University of Medicine and Pharmacy, 300041 Timisoara, Romania; 5 Research Center of the Institute of Cardiovascular Diseases Timisoara, 300310 Timisoara, Romania

**Keywords:** HDL functionality, small dense low-density lipoprotein (sdLDL), atherogenic risk, modern lipid-targeted therapy

## Abstract

High-density lipoprotein (HDL) and small dense low-density lipoprotein (sdLDL) represent two critical yet contrasting components in lipid metabolism and cardiovascular risk modulation. While HDL has traditionally been viewed as cardioprotective due to its role in reverse cholesterol transport and anti-inflammatory effects, emerging evidence emphasizes that HDL functionality—rather than concentration alone—is pivotal in atheroprotection. Conversely, sdLDL particles are increasingly recognized as highly atherogenic due to their enhanced arterial penetration, oxidative susceptibility, and prolonged plasma residence time. This review critically examined the physiological roles, pathological implications, and therapeutic interventions targeting HDL function and sdLDL burden. Lifestyle modifications, pharmacologic agents including statins, fibrates, PCSK9 inhibitors, and novel therapies such as icosapent ethyl were discussed in the context of their effects on HDL quality and sdLDL reduction. Additionally, current clinical guidelines were analyzed, highlighting a paradigm shift away from targeting HDL-C levels toward apoB-driven risk reduction. Although HDL-targeted therapies remain under investigation, the consensus supports focusing on lowering apoB-containing lipoproteins while leveraging lifestyle strategies to improve HDL functionality. In the setting of heart failure, particularly with preserved ejection fraction (HFpEF), alterations in HDL composition and elevated sdLDL levels have been linked to endothelial dysfunction and systemic inflammation, further underscoring their relevance beyond atherosclerosis. A comprehensive understanding of HDL and sdLDL dynamics is essential for optimizing cardiovascular prevention strategies.

## 1. Introduction

Cardiovascular disease (CVD) prevention strategies have traditionally prioritized the reduction of low-density lipoprotein cholesterol (LDL-C), often labeled as “bad cholesterol”, while high-density lipoprotein cholesterol (HDL-C) has long been regarded as “good cholesterol” due to its presumed protective effects. A consistent inverse association between HDL-C levels and CVD risk has been demonstrated in epidemiological studies, implicating low HDL-C as a marker of increased atherosclerotic burden. This evidence initially justified therapeutic approaches aimed at raising HDL-C levels [1]. Nevertheless, randomized controlled trials focused on pharmacologically elevating HDL-C have produced largely unsatisfactory outcomes. Agents such as niacin and cholesteryl ester transfer protein (CETP) inhibitors, although successful in increasing HDL-C concentrations, did not lead to a corresponding reduction in cardiovascular events [2,3]. Furthermore, Mendelian randomization studies have failed to confirm a causal link between elevated HDL-C and reduced cardiovascular risk, challenging the rationale for targeting HDL-C levels per se [4].

These findings have redirected scientific attention toward HDL functionality, particularly its role in reverse cholesterol transport (RCT). Cholesterol efflux capacity (CEC)—the ability of HDL to promote cholesterol removal from macrophages—has emerged as a more robust predictor of cardiovascular risk compared with HDL-C concentration alone [5,6,7].

In parallel, there has been growing recognition of the role of small dense LDL (sdLDL) particles, a particularly atherogenic LDL subfraction. Elevated sdLDL levels are strongly correlated with increased risk of atherosclerotic cardiovascular events, positioning sdLDL as a critical target for therapeutic modulation [8].

This review aimed to provide a comparative analysis of the cardiovascular implications of improving HDL functionality versus reducing sdLDL concentrations. Specifically, it evaluated their respective roles in atherogenesis, prognostic utility for cardiovascular risk assessment, potential therapeutic strategies, relevance to specific patient populations, and the current clinical consensus on managing these lipoprotein subfractions.

## 2. Biological Roles of HDL and sdLDL in Atherosclerosis

### 2.1. HDL: Reverse Cholesterol Transport and Atheroprotection

HDL plays a pivotal role in cardiovascular protection primarily through its involvement in RCT—a process that facilitates the mobilization of cholesterol from peripheral tissues, including macrophage foam cells within atherosclerotic plaques, to the liver for eventual excretion. Apolipoprotein A-I (apoA1), the principal apolipoprotein of HDL, initiates this process by accepting cholesterol from cells via the ATP-binding cassette transporter A1 (ABCA1). This is followed by the action of lecithin–cholesterol acyltransferase (LCAT), which esterifies free cholesterol, promoting the maturation of HDL particles capable of delivering cholesterol to hepatic and steroidogenic tissues [9].

ABCA1 serves as a critical regulator of both HDL biogenesis and cholesterol efflux, thereby contributing substantially to atheroprotection. Its overexpression in endothelial cells (ECs) not only enhances cholesterol efflux capacity but also exhibits anti-inflammatory effects, highlighting its multifaceted protective role [10]. LCAT further supports RCT by catalyzing the esterification of free cholesterol on HDL particles, a reaction that is potently activated by apoA1. This enzymatic modification promotes the structural remodeling and volumetric expansion of HDL, enhancing its cholesterol-carrying capacity [11].

In addition to mediating cholesterol transport, HDL possesses significant anti-inflammatory and antioxidant properties. In vitro experiments have shown that HDL inhibits the oxidative modification of LDL and attenuates endothelial activation, thereby reducing monocyte adhesion and chemokine secretion. Enzymes and proteins associated with HDL, such as paraoxonase-1 (PON1) and sphingosine-1-phosphate (S1P) bound to apolipoprotein M (apoM), actively scavenge oxidized lipids and stimulate endothelial nitric oxide (eNO) synthesis. These processes collectively support endothelial integrity and hinder the progression of atherosclerotic lesions. Functionally competent HDL is thus capable of extracting cholesterol from plaques, mitigating oxidative damage, and suppressing vascular inflammation [12,13].

### 2.2. HDL Structure and Function in Health vs. Disease

Pre-β HDL particles are synthesized through the lipidation of apolipoprotein A-I (apoA1) in an ATP-binding cassette transporter A1 (ABCA1)-dependent manner, occurring in the liver, intestine, and adipose tissue. These nascent HDL particles mature by incorporating cholesterol and phospholipids, subsequently delivering cholesterol to the liver via scavenger receptor class B type I (SR-BI) or exchanging it with apolipoprotein B (apoB)-containing lipoproteins through CETP-mediated mechanisms [14].

In healthy physiological states, HDL particles are phospholipid-rich and transport a range of bioactive molecules with anti-inflammatory and antioxidant properties, such as S1P and PON1, thereby facilitating RCT and stimulating eNO production. In contrast, dysfunctional HDL—commonly observed in metabolic disorders such as obesity and type 2 diabetes mellitus—tends to be enriched in triglycerides, depleted of phospholipids, and associated with pro-inflammatory proteins, including serum amyloid A (SAA). This dysfunctional phenotype is characterized by impaired cholesterol efflux and diminished antioxidant potential. Lifestyle interventions—such as structured physical activity, dietary modification, weight reduction, and bariatric surgery—have been shown to partially restore HDL functionality and composition [15,16,17].

Systemic inflammation and metabolic dysregulation may significantly compromise HDL’s protective functions. During acute inflammatory states, HDL undergoes compositional changes that attenuate its anti-inflammatory and antioxidant capabilities, potentially exacerbating vascular inflammation. In type 2 diabetes, HDL not only loses its capacity to promote cholesterol efflux but also fails to exert endothelial-protective effects. Enzymatic oxidation by agents such as myeloperoxidase further impairs HDL functionality by altering its structural integrity and inflammatory regulation. These findings support the growing consensus that HDL function, rather than its circulating concentration, plays a more pivotal role in modulating atherogenesis [18,19,20].

The failure of HDL-C-raising pharmacological agents to consistently reduce cardiovascular events has challenged the traditional emphasis on HDL quantity. Although niacin and fibrates can elevate HDL-C levels by 10–30%, the clinical benefit of such increases remains unproven. The CETP inhibitor torcetrapib, for example, raised HDL-C by approximately 70% but was associated with an unexpected increase in cardiovascular events, likely due to off-target effects [19]. Similarly, Mendelian randomization studies indicate that genetic variants linked to lifelong elevated HDL-C levels do not correlate with reduced atherosclerotic cardiovascular disease (ASCVD) risk [4].

Among various functional assays, cholesterol efflux capacity remains the most reliable measure of HDL function, showing a stronger inverse association with ASCVD than HDL-C levels alone. In a landmark study by Khera et al., macrophage cholesterol efflux capacity was significantly associated with reduced carotid intima-media thickness and angiographically confirmed coronary artery disease, independent of HDL-C concentrations [20]. Notably, individuals with comparable HDL-C levels exhibit wide variability in efflux capacity, with higher efflux consistently linked to lower cardiovascular risk, even after adjusting for HDL-C and apoA1 levels. Prospective data, including findings from the Dallas Heart Study, support the predictive value of HDL efflux for future cardiovascular events and all-cause mortality, while HDL-C levels lose statistical significance after multivariable adjustment [21]. Similarly, European cohort studies have reinforced the inverse association between cholesterol efflux and cardiovascular mortality, lending further support to the “dysfunctional HDL” hypothesis in risk stratification [22].

Although other aspects of HDL functionality—such as antioxidant and anti-inflammatory capacity—are under investigation as potential biomarkers, these measures currently lack standardized, clinically accessible assays. At present, cholesterol efflux capacity remains the most validated and clinically relevant functional assessment of HDL. Therefore, while low HDL-C levels should prompt comprehensive risk factor management, it is the functional integrity of HDL—its ability to mediate RCT and attenuate vascular inflammation—that is increasingly recognized as central to cardiovascular protection. This evolving perspective has catalyzed efforts to develop clinical assays for HDL function and novel therapies aimed at enhancing HDL activity, rather than merely increasing plasma HDL-C concentrations.

### 2.3. sdLDL: Highly Atherogenic Lipoprotein

Low-density lipoprotein (LDL) particles are not homogeneous in their atherogenic potential. Among the various LDL subfractions, sdLDL particles are considered markedly more atherogenic due to their unique physicochemical properties. Their smaller diameter and higher density enable more efficient penetration of the arterial intima, where they exhibit enhanced binding affinity to subendothelial proteoglycans. Furthermore, sdLDL particles are particularly susceptible to oxidative modification, attributable to their relatively low antioxidant content and increased surface area exposure. Oxidized sdLDL plays a pivotal role in foam cell formation and atheroma development.

A defining feature of sdLDL is its prolonged plasma half-life, driven by a reduced affinity for LDL receptors. While larger LDL particles typically circulate for approximately 40 h, sdLDL particles may persist for up to 74 h, thereby increasing their exposure to oxidative environments and enhancing their potential to infiltrate the endothelium. This triad of enhanced arterial penetration, delayed clearance, and oxidative susceptibility underlies the high atherogenic potential of sdLDL particles.

The predominance of sdLDL, commonly referred to as “LDL pattern B,” is associated with an elevated risk of coronary artery disease, even when total LDL-C concentrations are comparable to those with larger LDL particles. This suggests that LDL particle quality, rather than quantity alone, drives cardiovascular risk. Importantly, sdLDL elevation frequently coexists with other features of the “atherogenic dyslipidemia” characteristic of metabolic syndrome—namely, elevated triglycerides and reduced HDL-C levels—contributing synergistically to the development of premature ASCVD.

Disentangling the independent contribution of sdLDL to ASCVD risk has proven challenging, given its strong association with other lipid abnormalities and metabolic syndrome components. These interdependencies complicate statistical modeling, though numerous studies have attempted to clarify whether sdLDL is an independent risk predictor [8,23].

A comprehensive meta-analysis published in 2020, encompassing 21 prospective studies, found a consistent association between elevated sdLDL concentrations and increased cardiovascular event risk. Adjusted hazard ratios ranged from 1.21 to 1.52, depending on the method of sdLDL quantification and covariate adjustments [24].

Furthermore, sdLDL cholesterol (sdLDL-C) has demonstrated superior predictive value for ASCVD outcomes compared with total LDL-C or non-HDL-C, particularly among individuals with type 2 diabetes or stable coronary artery disease. In statin therapy trials, sdLDL-C remained a significant predictor of residual cardiovascular risk at low statin doses, but this association diminished at higher doses—likely due to a more profound reduction in sdLDL-C concentrations [25,26].

## 3. Interventions to Modify Quantity or Quality of HDL or sdLDL

### 3.1. Therapeutic Interventions to Raise HDL or Enhance HDL Function

#### 3.1.1. Lifestyle Interventions and HDL Modulation

Lifestyle modification remains a foundational strategy for enhancing both HDL concentrations and its atheroprotective functionality. Regular aerobic exercise and intentional weight reduction have been consistently shown to modestly increase HDL levels—typically in the range of 5–10%—while simultaneously improving HDL metabolic processing and functional efficacy. Physical activity enhances HDL’s cholesterol efflux capacity and antioxidant potential, partly by increasing the concentration of pre-β HDL particles and upregulating the activity of PON1, a key HDL-associated antioxidant enzyme.

Dietary patterns exert a significant influence on HDL dynamics. Diets low in refined carbohydrates and enriched with unsaturated fats—such as those found in nuts, olive oil, and fatty fish—are positively associated with increased HDL concentrations and enhanced HDL functionality. In contrast, high-carbohydrate diets have been linked to lower HDL levels and the formation of dysfunctional, triglyceride-enriched HDL particles, which exhibit diminished protective capacity [27,28,29].

Diet has a powerful influence not only on HDL cholesterol levels but also on the functionality and quality of HDL particles. Olive oil contributes to HDL health through its unique combination of monounsaturated fats and polyphenolic compounds. Its primary fatty acid, oleic acid, has been shown to modestly increase HDL cholesterol levels and enhance the stability of HDL particles. In addition, it supports the hepatic production of apolipoprotein A-I (ApoA-I), the main protein component of HDL, and may reduce the activity of enzymes such as cholesteryl ester transfer protein (CETP), which are involved in HDL catabolism [30,31,32].

Even more compelling is the role of olive oil polyphenols, such as hydroxytyrosol and oleuropein, which are abundant in extra virgin olive oil (EVOO). These bioactive compounds improve HDL functionality by promoting cholesterol efflux from macrophages—a crucial early step in reversing plaque formation. They also increase the activity of paraoxonase-1 (PON1), an enzyme associated with HDL that protects both HDL and LDL from oxidative damage. Additionally, polyphenols help preserve the anti-inflammatory capacity of HDL by preventing the incorporation of pro-inflammatory proteins such as serum amyloid A [33,34]

Several clinical studies have reinforced these findings. For example, the PREDIMED study demonstrated that a Mediterranean diet rich in EVOO not only raised HDL levels but also significantly enhanced HDL’s functional capacity, leading to a reduced risk of cardiovascular events. Other research has shown that individuals consuming EVOO exhibit higher antioxidant activity and better cholesterol efflux capacity compared to those consuming refined oils or oils rich in polyunsaturated fats. In summary, olive oil—especially in its extra virgin form—exerts multiple beneficial effects on HDL. By both modestly increasing HDL cholesterol levels and substantially enhancing HDL functionality, EVOO contributes meaningfully to cardiovascular protection [35,36]

Smoking cessation constitutes another crucial lifestyle intervention. Tobacco use has been shown to impair both HDL concentration and function, attenuating its anti-inflammatory properties and protective effects on endothelial function. As a result, current clinical guidelines strongly recommend smoking cessation alongside other lifestyle measures in individuals with low HDL levels, with the dual aim of modestly elevating HDL concentrations and restoring its vascular-protective properties.

Although the absolute rise in HDL concentration achieved through lifestyle modifications may be relatively modest, the overall cardiometabolic benefits are substantial. These interventions contribute to reduced plasma triglyceride levels, improved insulin sensitivity, and decreased concentrations of sdLDL particles, thereby reinforcing HDL’s role in cardiovascular protection [27,28,29].

#### 3.1.2. Pharmacologic Strategies Targeting HDL-C: Clinical Trials and Limitations

Efforts to pharmacologically elevate HDL-C have garnered significant attention; however, clinical outcomes have often failed to meet expectations. Niacin (vitamin B3) was one of the earliest agents shown to increase HDL-C levels, typically by 15–30%, while also reducing triglycerides and lipoprotein(a) [Lp(a)] concentrations. Despite these favorable lipid modifications, major clinical trials conducted in the context of statin therapy have not demonstrated incremental cardiovascular benefits [2,3].

The AIM-HIGH trial (2011), which evaluated extended-release niacin in patients with established cardiovascular disease already receiving statin therapy, reported a rise in HDL-C from approximately 35 to 42 mg/dL. However, this increase did not translate into a reduction in cardiovascular events compared with statin therapy alone. The trial was terminated early due to futility and noted a concerning increase in ischemic stroke incidence among niacin-treated participants [37]. Similarly, the HPS2-THRIVE trial (2014), encompassing over 25,000 high-risk individuals, investigated the combination of niacin with laropiprant. Although modest improvements in HDL (~6 mg/dL) and LDL (~10 mg/dL) were observed, there was no significant reduction in vascular event rates.

Moreover, niacin use was associated with a range of adverse effects, including flushing, hepatotoxicity, infections, impaired glucose tolerance, and increased bleeding risk [38].

Consequently, niacin is no longer endorsed by current clinical guidelines for routine management of residual cardiovascular risk and is now reserved for select cases, such as statin intolerance or severe elevations in Lp(a).

An alternative pharmacologic approach has involved targeting CETP, an enzyme that facilitates the exchange of cholesterol esters from HDL to apoB-containing lipoproteins in exchange for triglycerides. CETP inhibitors substantially raise HDL-C levels and modestly lower LDL-C. Despite their lipid-altering properties, clinical trials of four CETP inhibitors have largely yielded disappointing outcomes.

Torcetrapib, the first CETP inhibitor studied, increased HDL-C by more than 70% but was associated with elevated mortality and cardiovascular event rates in the ILLUMINATE trial, primarily due to off-target effects such as elevated blood pressure and aldosterone levels [39]. Subsequent CETP inhibitors—dalcetrapib and evacetrapib—avoided these off-target effects but still failed to improve cardiovascular outcomes in the dal-OUTCOMES and ACCELERATE trials, respectively, despite significant increases in HDL-C [40,41].

A more promising outcome was observed with anacetrapib in the REVEAL trial (2017). Anacetrapib nearly doubled HDL-C (~100%) and reduced LDL-C by approximately 17 mg/dL, leading to a statistically significant 9% reduction in major coronary events over a four-year period [42]. Nevertheless, the observed benefit appeared to be primarily attributable to LDL-C reduction, with the substantial HDL-C elevation having minimal independent impact. Furthermore, safety concerns related to long-term drug accumulation in adipose tissue ultimately led to the discontinuation of anacetrapib’s development.

Collectively, these findings underscore a pivotal realization in lipoprotein-targeted therapy: pharmacologically increasing HDL-C does not inherently confer cardiovascular protection. The functional quality of HDL, rather than its plasma concentration, is critical. As such, the reduction of atherogenic apoB-containing lipoproteins remains the most consistently effective strategy for cardiovascular risk reduction.

#### 3.1.3. Emerging and Adjunct Therapies Targeting HDL

In addition to statins and niacin, relatively few pharmacological agents exert direct effects on HDL. Fibrates, primarily used for lowering triglyceride concentrations, also increase HDL-C levels by approximately 10%. Clinical trials such as the Veterans Affairs High-Density Lipoprotein Intervention Trial (VA-HIT), which investigated gemfibrozil, and the Fenofibrate Intervention and Event Lowering in Diabetes (FIELD) trial, demonstrated modest HDL-C elevation accompanied by reductions in cardiovascular event rates—particularly in individuals presenting with atherogenic dyslipidemia, characterized by high triglycerides and low HDL-C levels [43,44]. Fibrates have additionally been reported to increase HDL particle size and may enhance HDL functionality, including improved reverse cholesterol transport (RCT) [45].

However, the efficacy of combining fibrates with statins remains uncertain. The ACCORD-Lipid trial, which examined the addition of fenofibrate to simvastatin therapy, did not show an overall reduction in cardiovascular events. Notably, a predefined subgroup analysis identified potential benefit in individuals with elevated triglycerides and low HDL-C, suggesting that fibrate therapy may have utility in this specific metabolic phenotype [46].

Thiazolidinediones (TZDs), such as pioglitazone—used primarily in the treatment of type 2 diabetes—have also been shown to modestly raise HDL-C levels and enhance HDL functionality, particularly by improving cholesterol efflux capacity. However, their clinical use in lipid management is limited due to their primary indication for glycemic control and associated adverse effect profile [47].

A novel therapeutic direction involves targeting HDL functionality rather than its plasma concentration. This includes infusion-based therapies utilizing reconstituted HDL particles or apolipoprotein A-I (apoA-I) mimetic peptides, such as apoA-I Milano. Early-phase clinical trials have demonstrated that these agents can augment cholesterol efflux capacity and may even induce regression of atherosclerotic plaque volume as assessed by imaging techniques [48]. One such agent, CSL112—a plasma-derived, reconstituted apoA-I infusion—is currently under investigation in a Phase 3 clinical trial to evaluate its efficacy in reducing recurrent cardiovascular events when administered acutely after myocardial infarction [49]. These emerging therapies reflect a strategic shift toward enhancing HDL function rather than sustaining elevated HDL-C levels.

Collectively, these developments reinforce a critical paradigm shift in lipidology: pharmacologically raising HDL-C alone does not reliably translate into cardiovascular benefit, particularly when LDL-C is effectively managed. The emphasis is increasingly placed on optimizing HDL functionality—its ability to facilitate cholesterol efflux, exert antioxidant and anti-inflammatory effects, and support endothelial health. Nevertheless, the cornerstone of ASCVD risk reduction remains the lowering of atherogenic apoB-containing lipoproteins, including LDL-C and non-HDL-C, which continue to demonstrate the most consistent and evidence-based clinical benefit.

### 3.2. Targeting sdLDL Particles: Interventions and Outcomes

Given the highly atherogenic potential of sdLDL particles, interventions aimed at reducing sdLDL concentrations or shifting LDL particle distribution toward larger, less dense forms are clinically desirable. Notably, several established lipid-lowering therapies have been shown to disproportionately benefit the sdLDL subfraction.

#### 3.2.1. Statins

HMG-CoA reductase inhibitors, the first-line agents for lowering LDL-C, are supported by extensive evidence demonstrating their efficacy in reducing cardiovascular risk. Statins enhance hepatic LDL receptor expression, thereby increasing the clearance of LDL particles of all sizes. This action often induces a phenotypic shift from small, dense to larger LDL particles as the overall LDL burden decreases. High-intensity statin therapy has been shown to significantly reduce sdLDL particle concentrations. For instance, rosuvastatin at 40 mg daily achieves greater reductions in sdLDL levels compared with lower doses. Mechanistically, statins reduce hepatic very low-density lipoprotein (VLDL) production—an upstream precursor of sdLDL—and enhance LDL receptor recycling, thus facilitating sdLDL clearance. Although residual sdLDL may persist in patients with hypertriglyceridemia, statins remain the cornerstone of therapy, lowering apoB-containing lipoproteins, including sdLDL, and conferring a 20–30% risk reduction in ASCVD per mmol/L LDL-C reduction [50].

#### 3.2.2. Fibrates

Fibric acid derivatives, such as fenofibrate and gemfibrozil, are particularly effective in individuals with elevated triglycerides and a predominance of small LDL particles. By activating peroxisome proliferator-activated receptor-alpha (PPAR-α), fibrates upregulate lipoprotein lipase (LPL) activity and enhance fatty acid oxidation, thereby reducing VLDL production and promoting HDL synthesis. This mechanism limits the substrate availability for sdLDL formation. Clinical data support fibrates’ benefit in sdLDL-predominant dyslipidemia. In the VA-HIT trial, gemfibrozil therapy reduced coronary artery disease events by 22% in men with low HDL. Similarly, the ACCORD-Lipid trial revealed no overall benefit from adding fenofibrate to statin therapy, but subgroup analyses indicated a 30% reduction in events among patients with triglycerides >200 mg/dL and HDL <35 mg/dL. A post hoc analysis concluded that fenofibrate is particularly beneficial in diabetic patients with mixed dyslipidemia—those most likely to harbor elevated sdLDL. Fibrates may also modestly improve HDL particle size. However, caution is warranted when co-administered with statins due to myopathy risk; fenofibrate is generally safer than gemfibrozil in combination therapy [46,51].

#### 3.2.3. Niacin

Niacin exerts multifaceted lipid effects, including triglyceride reduction (20–50%) and HDL-C elevation. It can induce a phenotypic shift from LDL pattern B (sdLDL) to pattern A (large buoyant LDL). Early angiographic trials demonstrated niacin’s capacity to slow plaque progression, potentially via sdLDL reduction. However, in contemporary large-scale trials conducted alongside statin therapy, niacin failed to show incremental benefit and was associated with significant adverse effects. While niacin may still reduce sdLDL concentrations, its unfavorable risk–benefit profile and lack of additive efficacy in well-treated patients have led to a general recommendation against its routine use [37].

#### 3.2.4. Proprotein Convertase Subtilisin/Kexin Type 9 (PCSK9) Inhibitors

Monoclonal antibodies targeting PCSK9, such as alirocumab and evolocumab, significantly reduce LDL particle concentrations by preventing degradation of LDL receptors. This results in increased hepatic clearance of apoB-containing lipoproteins, including sdLDL. Clinical trials such as FOURIER and ODYSSEY OUTCOMES have shown that PCSK9 inhibitors reduce LDL-C by 50–60% and confer an additional 15–20% risk reduction in high-risk populations on statin background therapy. These agents not only lower circulating LDL but may also reduce the hepatic production of new LDL particles. PCSK9 inhibitors are particularly valuable in patients with persistent sdLDL elevation, such as those with familial hypercholesterolemia or metabolic syndrome, where conventional therapy is insufficient. Despite their high cost, these agents are effective tools for minimizing sdLDL burden in select high-risk patients [51,52].

#### 3.2.5. Omega-3 Fatty Acids

Pharmaceutical-grade omega-3 fatty acids, particularly high-dose eicosapentaenoic acid (EPA), have been shown to lower triglycerides by 25–50%, thereby potentially reducing sdLDL formation. In the REDUCE-IT trial (2018), administration of 4 g/day of EPA resulted in a 25% relative risk reduction in major cardiovascular events among patients with elevated triglycerides (200–500 mg/dL) already on statin therapy. Proposed mechanisms include triglyceride lowering, plaque stabilization, and anti-inflammatory effects. Omega-3 therapy may also promote a shift toward larger LDL particles, although EPA can sometimes raise total LDL-C by converting small, dense LDL into larger, cholesterol-rich particles. Importantly, the cardiovascular benefit observed in REDUCE-IT was independent of baseline LDL-C, indicating that EPA targeted residual risk linked to triglyceride-rich lipoproteins and sdLDL. Icosapent ethyl is now guideline-recommended for individuals with ASCVD or diabetes and elevated triglycerides. Over-the-counter fish oil supplements are not equivalent to prescription EPA, and DHA-containing mixtures may paradoxically raise LDL [53].

#### 3.2.6. Other Interventions

Lifestyle modifications—particularly weight loss and dietary adjustments—remain critical for lowering sdLDL concentrations. Reducing simple carbohydrate intake and achieving weight loss significantly diminishes hepatic triglyceride synthesis and sdLDL production. Diets emphasizing unsaturated fats over carbohydrates or saturated fats favor larger LDL particle formation, while high-carbohydrate, low-fat regimens may paradoxically elevate sdLDL despite reducing LDL-C. Glycemic control is also pivotal: poorly controlled diabetes promotes LDL glycation, impairs clearance, and facilitates sdLDL accumulation. Emerging therapies such as bempedoic acid, a novel oral agent approved for statin-intolerant patients, have demonstrated modest ASCVD risk reduction in the CLEAR Outcomes trial. Although bempedoic acid does not specifically target sdLDL, any effective reduction in apoB-containing lipoproteins contributes to lowering sdLDL levels [54,55].

In summary, most therapies that reduce triglycerides or apoB-containing particles also favorably impact sdLDL concentrations. Statins remain the cornerstone of treatment and are frequently complemented by fibrates or omega-3 fatty acids in individuals with residual hypertriglyceridemia. While agents like niacin and CETP inhibitors can lower sdLDL mechanistically, they have not demonstrated outcome benefits and are not routinely recommended. Ultimately, reducing the total atherogenic particle burden—reflected by non-HDL-C or apoB—is the primary therapeutic objective. When sdLDL is elevated, as in metabolic syndrome, addressing the underlying metabolic disturbances (e.g., insulin resistance and VLDL overproduction) is more clinically relevant than targeting sdLDL in isolation.

## 4. Special Populations and Considerations

### 4.1. Dyslipidemia in Metabolic Syndrome and Type 2 Diabetes Mellitus: Pathophysiology and Therapeutic Strategies

Individuals with metabolic syndrome (MetS) or type 2 diabetes mellitus (T2DM) frequently exhibit a distinct lipid profile known as atherogenic dyslipidemia, characterized by elevated triglycerides, reduced HDL-C, and a predominance of sdLDL particles. This constellation significantly accelerates atherosclerosis and contributes to increased cardiovascular risk. Notably, despite normal or near-normal LDL-C levels, LDL particles in this population are often smaller and denser, thereby retaining high atherogenic potential. Additionally, HDL particles may be functionally impaired due to chronic hyperglycemia-induced glycation and systemic inflammation, which diminish their antioxidant and anti-inflammatory properties.

#### 4.1.1. Lifestyle Modification

Weight reduction achieved through dietary changes and regular physical activity is foundational in managing atherogenic dyslipidemia. These interventions reduce hepatic very-low-density lipoprotein (VLDL) synthesis, elevate HDL-C levels, and promote a phenotypic shift toward larger, less atherogenic LDL particles. Low-carbohydrate and Mediterranean-style dietary patterns have demonstrated particular efficacy in lowering triglycerides and improving HDL-C concentrations.

#### 4.1.2. Statin Therapy

Statins remain a cornerstone of lipid management in patients with T2DM and MetS. For individuals aged 40 years and older with T2DM, statin therapy is recommended regardless of baseline LDL-C levels, owing to its capacity to lower apolipoprotein B (apoB) levels and reduce the number of circulating atherogenic particles. Statins are equally integral to primary prevention in patients with MetS, given their robust effect on cardiovascular risk reduction.

#### 4.1.3. Fibrate Therapy

For patients with persistent hypertriglyceridemia (≥200 mg/dL) and low HDL-C (≤34 mg/dL) despite statin therapy, adjunctive treatment with fenofibrate may be beneficial. The ACCORD-Lipid trial suggested that the addition of fenofibrate to statin therapy reduced cardiovascular event rates in this dyslipidemic subgroup. Moreover, fenofibrate has demonstrated microvascular benefits, such as attenuating the progression of diabetic retinopathy.

#### 4.1.4. Icosapent Ethyl (EPA)

High-dose icosapent ethyl (4 g/day) has shown cardiovascular benefit in patients with T2DM and elevated triglycerides (≥150 mg/dL) already receiving statin therapy. The REDUCE-IT trial reported a significant reduction in ischemic cardiovascular events in this population, supporting the utility of EPA supplementation as an adjunctive therapy in high-risk individuals.

#### 4.1.5. Niacin

Although niacin can favorably modify lipid profiles by increasing HDL-C and reducing triglycerides, large clinical trials such as AIM-HIGH and HPS2-THRIVE have not demonstrated additional cardiovascular benefit when niacin is added to statin therapy in patients with T2DM or MetS. Additionally, niacin may impair glycemic control, limiting its use in this population.

#### 4.1.6. ApoB and Non-HDL Cholesterol Assessment

Given the frequent discordance between LDL-C concentrations and atherogenic particle burden in MetS and T2DM, measurement of apoB or non-HDL cholesterol offers a more accurate estimation of cardiovascular risk. Elevated apoB levels, even in the presence of normal LDL-C, indicate increased numbers of atherogenic lipoproteins and may warrant intensified lipid-lowering therapy.

Effective management of dyslipidemia in patients with MetS and T2DM requires a multifaceted approach that prioritizes lifestyle intervention and statin therapy. In selected patients, adjunctive use of fibrates or icosapent ethyl may be indicated. Measurement of apoB and non-HDL cholesterol can further refine cardiovascular risk stratification and guide therapeutic decision-making [38,46,53,54,56,57].

### 4.2. Management of Dyslipidemia in Statin-Intolerant Patients

The management of patients who are intolerant to statin therapy remains a significant clinical challenge, particularly given their persistent elevation of LDL-C and the frequent presence of atherogenic sdLDL particles. In such individuals, alternative strategies are essential for reducing the burden of apoB-containing lipoproteins and mitigating residual cardiovascular risk.

#### 4.2.1. Ezetimibe

As a selective cholesterol absorption inhibitor, ezetimibe reduces LDL-C by approximately 15–20%. The IMPROVE-IT trial demonstrated that the addition of ezetimibe to statin therapy resulted in further LDL-C reduction and improved cardiovascular outcomes, supporting its use in combination or as monotherapy in statin-intolerant individuals [58].

#### 4.2.2. PCSK9 Inhibitors

Monoclonal antibodies targeting proprotein convertase subtilisin/kexin type 9 (PCSK9), such as alirocumab and evolocumab, offer substantial LDL-C reductions exceeding 50%. These agents effectively lower circulating apoB concentrations, including sdLDL, and are particularly valuable in statin-intolerant patients or those with very high baseline cardiovascular risk [59].

#### 4.2.3. Bempedoic Acid

This novel oral agent inhibits ATP-citrate lyase, an upstream enzyme in the cholesterol synthesis pathway, leading to LDL-C reductions of approximately 20%. In patients with documented statin intolerance, bempedoic acid has demonstrated efficacy in reducing major adverse cardiovascular events, offering an important oral alternative [55].

#### 4.2.4. Lipoprotein Apheresis

For individuals with familial hypercholesterolemia and statin intolerance, lipoprotein apheresis may be considered. This extracorporeal procedure acutely removes LDL-C, lipoprotein(a), and sdLDL particles from the circulation. Due to its invasive nature and cost, it is typically reserved for patients at very high cardiovascular risk who are refractory to pharmacologic therapy [60].

#### 4.2.5. Niacin and Fibrates

##### Niacin

Although niacin has been shown to raise HDL-C and lower triglycerides, its clinical utility in statin-intolerant patients is limited. In earlier trials, such as the Coronary Drug Project, niacin monotherapy reduced recurrent myocardial infarction. However, its combination with statins in contemporary studies has failed to yield additional benefit and has been associated with adverse metabolic effects, including impaired glycemic control [61].

##### Fibrates

Primarily indicated for hypertriglyceridemia, fibrates are effective in reducing triglyceride concentrations and the risk of pancreatitis in patients with triglyceride levels ≥500 mg/dL. Additionally, they may confer modest cardiovascular benefit in patients with elevated apoB concentrations, although their use must be balanced against potential adverse effects, especially when used concomitantly with statins [62].

#### 4.2.6. Combination Strategies

Comprehensive risk factor management remains essential. Optimizing blood pressure, glycemic control, nutrition, and physical activity has a profound impact on cardiovascular outcomes. Some patients previously labeled as statin-intolerant may tolerate lower doses or intermittent statin regimens. In confirmed intolerance, combination therapy—such as ezetimibe with bempedoic acid or PCSK9 inhibitors—can effectively reduce LDL-C and sdLDL levels to target goals.

In statin-intolerant individuals, therapeutic focus should be directed toward reducing the overall atherogenic particle burden—primarily apoB-containing lipoproteins—rather than solely attempting to raise HDL-C. The availability of newer lipid-lowering agents provides a robust and expanding toolkit for managing dyslipidemia in this high-risk population [55,58,59,60,61,62,63].

Table 1 provides a comparative overview of key lipid-lowering therapies, summarizing their mechanisms of action, recommended dosages, notable drug interactions, and supporting clinical trial outcomes. This table serves as a concise reference for evaluating the pharmacologic strategies used in dyslipidemia management. Table 2 complements this by detailing the side effect profiles and demographic considerations of the same drug classes, including variability related to dosage, race, sex, and body mass index (BMI). Together, these tables offer a comprehensive snapshot of efficacy, safety, and patient-specific factors relevant to therapeutic decision-making.

Various dietary and pharmacological interventions modulate lipoprotein metabolism, particularly impacting HDL functionality and sdLDL levels. Foods rich in monounsaturated fats and polyphenols, such as extra virgin olive oil, nuts, and red wine, stimulate ApoA-I synthesis and activate the cholesterol transporters ABCA1 and ABCG1, thereby enhancing cholesterol efflux from macrophages. These particles are further esterified by LCAT, leading to the formation of mature HDL with improved antioxidant and anti-inflammatory capacity [30,31,32,33,34]. Additionally, low-carbohydrate diets and omega-3 fatty acids (EPA) reduce triglyceride levels and hepatic VLDL production, limiting the conversion to sdLDL [28,29,53]. Pharmacologically, statins and PCSK9 inhibitors upregulate hepatic LDL receptor (LDLR) expression, increasing the clearance of apoB-containing lipoproteins, including sdLDL [50,51,52]. Fibrates activate PPAR-α, promoting lipoprotein lipase activity, reducing VLDL production, and enhancing HDL particle composition [43,44,45,46,51]. Ezetimibe, by inhibiting intestinal cholesterol absorption via NPC1L1, reduces hepatic cholesterol influx and stimulates LDLR expression [55], while bempedoic acid inhibits ATP-citrate lyase, decreasing hepatic cholesterol synthesis with similar effects [55,56]. Furthermore, omega-3 fatty acids have been shown to significantly reduce cardiovascular risk by lowering triglycerides, limiting sdLDL formation, and exerting anti-inflammatory effects [53].

**Figure 1:**
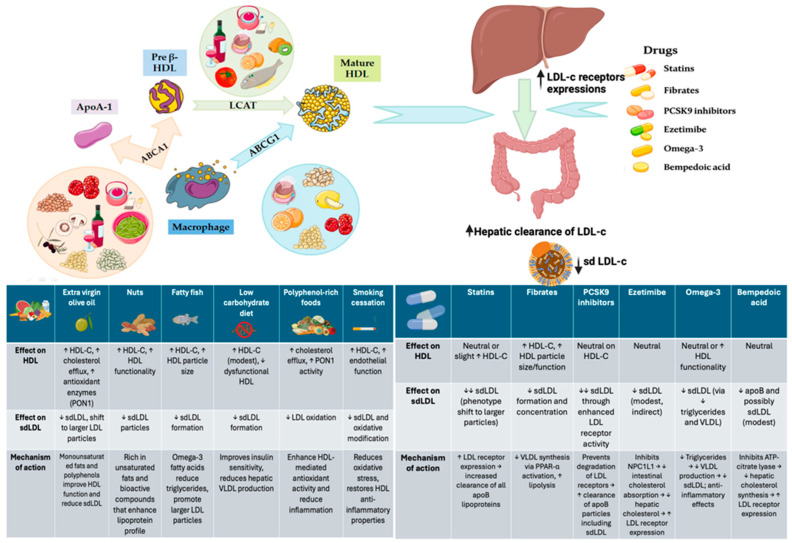
Modulation of HDL functionality and sdLDL levels through dietary and pharmacological interventions; HDL—high-density lipoprotein cholesterol; LDL—low-density llipoprotein cholesterol; sdLDL—small dense low-density lipoprotein cholesterol; ApoA-I—apolipoprotein A-I; ABCA1—ATP-binding cassette transporter A1; ABCG1—ATP-binding cassette transporter G1; LCAT—lecithin–cholesterol acyltransferase; PON1—paraoxonase-1; PCSK9—proprotein convertase subtilisin/kexin type 9; NPC1L1—niemann–pick C1-like 1 transporter; PPAR-α—peroxisome proliferator-activated receptor alpha; RCT—reverse cholesterol transport; EPA—eicosapentaenoic acid; DHA—docosahexaenoic acid; TG—triglycerides; VLDL—very low-density lipoprotein.

Figure 1 provides a schematic overview illustrating the mechanisms of action by which various pharmacological agents and dietary components influence small dense LDL cholesterol (sdLDL-C) and high-density lipoprotein cholesterol (HDL-C) metabolism.

## 5. Clinical Guidelines and Consensus Perspective

Contemporary clinical guidelines consistently prioritize the reduction of atherogenic lipoproteins—specifically LDL-C and non-high-density lipoprotein cholesterol (non-HDL-C)—as the primary strategy for ASCVD prevention. Both the American College of Cardiology/American Heart Association (ACC/AHA) and the European Society of Cardiology/European Atherosclerosis Society (ESC/EAS) guidelines advise against directly targeting HDL-C levels, citing the lack of evidence demonstrating cardiovascular benefit from pharmacologically increasing HDL-C concentrations. The 2018 ACC/AHA guideline explicitly states that HDL-C should not be used as a treatment target. Similarly, the 2019 ESC/EAS guidelines assert that pharmacologic elevation of HDL-C has not yielded favorable outcomes and caution that extremely high HDL-C levels may not confer additional protection [70,71].

Although HDL functionality markers, such as cholesterol efflux capacity, hold prognostic value, these assessments are not yet standardized for clinical use and remain confined to research settings. Expert consensus suggests that, with further validation, these assays may enhance risk stratification and aid in evaluating the efficacy of emerging therapies [21,72].

In contrast, current guidelines do not advocate for the routine measurement of LDL subfractions such as small dense sdLDL. Instead, apoB or non-HDL-C are endorsed as practical surrogate markers for assessing total atherogenic particle burden, particularly in patients with elevated triglycerides, metabolic syndrome, type 2 diabetes, or obesity, all of which are associated with increased sdLDL concentrations. The 2019 ESC/EAS guidelines recommend apoB measurement in individuals exhibiting features of metabolic dyslipidemia, while the ACC/AHA guidelines regard apoB and LDL particle counts as optional “risk-enhancing” factors, recognizing that cost and availability limit their widespread implementation. In cases of hypertriglyceridemia (>200 mg/dL), non-HDL-C is typically used as a secondary treatment target, with goals of <100 mg/dL for high-risk individuals, as it more comprehensively reflects risk from sdLDL and remnant particles than LDL-C alone.

Universal across all major guidelines is the endorsement of lifestyle modification as the foundational approach for managing dyslipidemia. Specifically, exercise, dietary intervention, and weight management are strongly recommended for improving HDL-C and reducing sdLDL, while pharmacologic HDL-C-raising agents such as niacin are not advised. In patients with controlled LDL-C but persistently low HDL-C, clinicians are encouraged to focus on other modifiable risk factors (e.g., blood pressure, smoking cessation) rather than targeting HDL pharmacologically. This recommendation is supported by trial data; for example, the AIM-HIGH trial found no additional benefit from niacin in patients on optimal statin therapy.

Recent guideline updates have integrated findings from the REDUCE-IT trial. For patients with residual dyslipidemia characterized by elevated triglycerides and likely sdLDL enrichment, the addition of icosapent ethyl to statin therapy is now recommended. The 2019 ACC/AHA guideline assigns a Class IIa recommendation to icosapent ethyl for patients with established ASCVD, triglycerides ≥150 mg/dL, and controlled LDL-C, as this strategy mitigates risk from remnant and sdLDL particles without necessitating direct measurement.

Expert consensus continues to emphasize that apoB-containing lipoproteins—including LDL, sdLDL, VLDL, and lipoprotein(a) [Lp(a)]—are the primary mediators of atherosclerosis. Accordingly, therapeutic strategies should aim to reduce apoB levels. HDL-C, in contrast, is considered a modulator of risk rather than a direct target; low HDL-C should prompt investigation of underlying metabolic disturbances such as insulin resistance, physical inactivity, or smoking, which can be addressed through non-pharmacologic means. While ongoing research into HDL-targeted therapies (e.g., recombinant apoA-I, cholesterol efflux enhancers) may eventually reshape therapeutic priorities, current guidelines remain firmly focused on reducing atherogenic lipoprotein burden [71,72,73].

Heart failure (HF), a major contributor to cardiovascular morbidity and mortality, is closely influenced by lipid metabolism abnormalities, including the balance between HDL and small dense LDL (sdLDL) particles [74]. Dyslipidemia not only accelerates atherosclerotic progression but also promotes systemic inflammation and endothelial dysfunction, key pathophysiological components in the development and progression of heart failure, particularly the ischemic subtype. Elevated levels of sdLDL have been implicated in microvascular dysfunction and impaired coronary perfusion, which can precipitate myocardial injury and contribute to ventricular remodeling [75].

Moreover, recent evidence suggests that HDL dysfunction—characterized by a loss of its antioxidative, anti-inflammatory, and endothelial-protective properties—may be a critical factor in heart failure pathogenesis. In patients with HF, particularly those with preserved ejection fraction (HFpEF), HDL particles often exhibit altered composition and reduced functionality. This impairment diminishes their capacity to mediate reverse cholesterol transport and to suppress inflammatory signaling pathways within the myocardium, potentially exacerbating cardiac fibrosis and stiffness [21,76].

From a therapeutic perspective, interventions that improve HDL function and reduce sdLDL burden may offer potential benefits in heart failure management. Statins, while primarily prescribed for atherosclerosis and LDL-C reduction, may exert pleiotropic effects that include improved endothelial function and attenuation of left ventricular hypertrophy. Similarly, omega-3 fatty acid formulations such as icosapent ethyl have shown promise in reducing cardiovascular events and inflammation, which may indirectly benefit patients with established HF or those at high risk [53,77].

Incorporating lipid quality metrics, such as apoB and HDL functionality, into risk stratification models may enhance clinical decision-making in heart failure populations. As research continues to uncover the intricate relationships between lipoproteins and myocardial health, personalized strategies that go beyond traditional cholesterol targets could help mitigate heart failure progression and improve outcomes [78].

## 6. Conclusions

Optimal cardiovascular risk reduction requires a comprehensive and individualized approach to dyslipidemia management. Although HDL possesses a range of atheroprotective properties—including reverse cholesterol transport, anti-inflammatory activity, and endothelial support—clinical trials have consistently demonstrated that pharmacologically increasing HDL-C in isolation does not reduce ASCVD event rates. Conversely, small, dense LDL particles are well established as highly atherogenic, and interventions that reduce total LDL particle burden, including sdLDL, have shown clear benefit.

Current lipid management strategies, therefore, emphasize aggressive lowering of LDL-C and apoB-containing lipoproteins through statins, ezetimibe, PCSK9 inhibitors, fibrates, and structured lifestyle modification. These approaches not only reduce sdLDL concentrations but also address broader residual risk. Meanwhile, evolving metrics of HDL functionality, such as cholesterol efflux capacity, may offer refined tools for cardiovascular risk assessment as evidence develops.

At present, clinicians should continue to follow evidence-based guidelines prioritizing LDL-C and apoB reduction, with secondary emphasis on improving HDL function through lifestyle measures. Addressing both sdLDL and HDL function within an integrated, apoB-centered strategy remains the most practical and effective method for mitigating atherosclerotic risk. As future therapies targeting HDL function mature and demonstrate outcome benefits, clinical practice guidelines will evolve accordingly. However, the fundamental principle endures: reducing the burden of atherogenic lipoproteins while leveraging HDL’s physiological roles remains the cornerstone of ASCVD prevention.

## Figures and Tables

**Table 1:** Comparative overview of lipid-lowering therapies: mechanisms, dosages, interactions, and clinical evidence.

Drug	Mechanism of Action	Recommended Dosage	Interactions	Key Clinical Trials and Outcomes
Statins	Inhibit HMG-CoA reductase, reducing hepatic cholesterol synthesis, upregulate LDL receptors	Atorvastatin 10–80 mg/day; Rosuvastatin 5–40 mg/day	CYP3A4 inhibitors (e.g., grapefruit, some antibiotics), fibrates ↑ myopathy risk	4S, HPS, ASCOT-LLA [64,65,66]: Showed significant reduction in LDL and cardiovascular events
Fibrates	Activate PPAR-α, increase fatty acid oxidation, decrease triglycerides, modest HDL increase	Fenofibrate 145 mg/day; Gemfibrozil 600 mg BID	Statins ↑ myopathy risk, anticoagulants ↑ bleeding	FIELD, ACCORD Lipid [44,46]: Showed triglyceride reduction; cardiovascular benefits in dyslipidemic subgroups
Niacin	Inhibits hepatic synthesis of VLDL, reduces LDL and TG, increases HDL by reducing hepatic uptake	Niacin 500–2000 mg/day (ER form)	Statins ↑ myopathy risk; antihypertensives may enhance flushing	AIM-HIGH, HPS2-THRIVE [37,38]: No added CV benefit when added to statins; increased side effects
PCSK9 Inhibitors	Inhibit PCSK9 binding to LDL receptors, increase LDL clearance	Alirocumab 75–150 mg SC every 2–4 weeks; Evolocumab 140 mg SC every 2 weeks	No major interactions; safe with statins	FOURIER, ODYSSEY OUTCOMES [51,52]: Significant LDL reduction and reduced cardiovascular events in high-risk patients
Ezetimibe	Inhibits NPC1L1 cholesterol transporter in the intestine, reduces cholesterol absorption	10 mg/day	Synergistic with statins, no CYP interactions	IMPROVE-IT [58]: Demonstrated incremental LDL-C lowering and event reduction when added to statin therapy
Omega-3 Fatty Acids	Reduce hepatic VLDL-TG synthesis and increase TG clearance, may raise HDL modestly	EPA/DHA 2–4 g/day	May enhance anticoagulant effect; careful with bleeding risk	REDUCE-IT (EPA), STRENGTH (EPA+DHA) [53,67]: REDUCE-IT showed cardiovascular benefit; STRENGTH did not
Bempedoic Acid	Inhibits ATP citrate lyase, reducing cholesterol synthesis upstream of HMG-CoA reductase	180 mg/day	Statins (no increased risk); gout meds due to uric acid elevation	CLEAR Harmony, CLEAR Wisdom [55,68,69]: Showed modest LDL-C reduction; well-tolerated as adjunct to statins

LDL—Low-Density Lipoprotein, HDL—High-Density Lipoprotein, TG—Triglycerides, VLDL—Very Low-Density Lipoprotein, PPAR-α—Peroxisome Proliferator-Activated Receptor Alpha, CYP3A4—Cytochrome P450 3A4, SC—Subcutaneous, EPA—Eicosapentaenoic Acid, DHA—Docosahexaenoic Acid, CV—Cardiovascular.

**Table 2:** Side effects and demographic considerations of lipid-lowering therapies.

Drug	Low Dose Side Effects	High Dose Side Effects	Race Considerations	Sex Considerations	BMI Considerations
Statins [65]	Generally well tolerated; mild myalgias	Myopathy, elevated liver enzymes, increased diabetes risk	Asians may require lower doses due to increased sensitivity	Equally effective; women may have higher rates of side effects	Obese patients may need higher doses; response preserved
Fibrates [44]	Mild GI upset, rash	Myopathy (esp. with statins), gallstones, liver enzyme elevation	No major race-specific considerations	Similar effects in both sexes	More effective in hypertriglyceridemic obese individuals
Niacin [37]	Flushing, GI discomfort	Hepatotoxicity, hyperglycemia, gout, severe flushing	Limited data; similar efficacy across groups	More flushing reported in women	Higher side effect risk with obesity
PCSK9 Inhibitors [51]	Injection site reactions	Rare neurocognitive effects, injection site pain	Effective across races; used in statin-intolerant populations	Effective in both sexes	Effective regardless of BMI
Ezetimibe [58]	Mild GI discomfort	Minimal; rare hepatic enzyme elevations	Consistent efficacy across races	No sex-based differences reported	No major impact of BMI
Omega-3 Fatty Acids [53]	Mild GI symptoms, fishy aftertaste	Increased bleeding risk, possible atrial fibrillation	Similar efficacy across races	No sex-based differences reported	Greater benefit in high-TG obese patients
Bempedoic Acid [68]	Generally well tolerated; mild muscle symptoms	Elevated uric acid, tendon rupture risk	Limited race-specific data; presumed similar efficacy	No significant differences observed	Efficacy maintained in obesity; uric acid monitoring important

GI—Gastrointestinal, BMI—Body Mass Index, TG—Triglycerides.

## Data Availability

Data sharing is not applicable.

## Abbreviations

The following abbreviations are used in this manuscript:

ACC/AHA	American College of Cardiology/American Heart Association
ACCORD	Action to Control Cardiovascular Risk in Diabetes
AIM-HIGH	Atherothrombosis Intervention in Metabolic Syndrome with Low HDL/High Triglycerides and Impact on Global Health Outcomes
ApoA-I	Apolipoprotein A-I
ApoB	Apolipoprotein B
ASCVD	Atherosclerotic Cardiovascular Disease
CEC	Cholesterol Efflux Capacity
CETP	Cholesteryl Ester Transfer Protein
CSL112	A reconstituted HDL/ApoA-I infusion therapy
DHA	Docosahexaenoic Acid
EPA	Eicosapentaenoic Acid
ESC/EAS	European Society of Cardiology/European Atherosclerosis Society
eNO	Endothelial Nitric Oxide
EVOO	extra virgin olive oil
FDA	Food and Drug Administration
FIELD	Fenofibrate Intervention and Event Lowering in Diabetes
FOURIER	Further Cardiovascular Outcomes Research with PCSK9 Inhibition in Subjects with Elevated Risk
HDL	High-Density Lipoprotein
HDL-C	High-Density Lipoprotein Cholesterol
HF	Heart Failure
HFpEF	Heart Failure with Preserved Ejection Fraction
HPS2-THRIVE	Heart Protection Study 2–Treatment of HDL to Reduce the Incidence of Vascular Events
IDL	Intermediate-Density Lipoprotein
ILLUMINATE	Investigation of Lipid Level Management to Understand its Impact in Atherosclerotic Events
IMPROVE-IT	Improved Reduction of Outcomes: Vytorin Efficacy International Trial
JACC	Journal of the American College of Cardiology
LCAT	Lecithin–Cholesterol Acyltransferase
LDL	Low-Density Lipoprotein
LDL-C	Low-Density Lipoprotein Cholesterol
Lp(a)	Lipoprotein(a)
MetS	Metabolic Syndrome
MPO	Myeloperoxidase
Non-HDL-C	Non–High-Density Lipoprotein Cholesterol
PCSK9	Proprotein Convertase Subtilisin/Kexin Type 9
PON1	Paraoxonase-1
RCT	Reverse Cholesterol Transport
REVEAL	Randomized Evaluation of the Effects of Anacetrapib Through Lipid-modification
REDUCE-IT	Reduction of Cardiovascular Events with Icosapent Ethyl–Intervention Trial
S1P	Sphingosine-1-Phosphate
SAA	Serum Amyloid A
sdLDL	Small Dense Low-Density Lipoprotein
sdLDL-C	Small Dense Low-Density Lipoprotein Cholesterol
SR-BI	Scavenger Receptor Class B Type I
T2DM	Type 2 Diabetes Mellitus
TZDs	Thiazolidinediones
VA-HIT	Veterans Affairs High-Density Lipoprotein Intervention Trial
VLDL	Very-Low-Density Lipoprotein
